# Human Tregs Made Antigen Specific by Gene Modification: The Power to Treat Autoimmunity and Antidrug Antibodies with Precision

**DOI:** 10.3389/fimmu.2017.01117

**Published:** 2017-09-21

**Authors:** Patrick R. Adair, Yong Chan Kim, Ai-Hong Zhang, Jeongheon Yoon, David W. Scott

**Affiliations:** ^1^Department of Medicine, Uniformed Services University of the Health Sciences, Bethesda, MD, United States

**Keywords:** human regulatory CD4^+^ T cells, Tregs, hemophilia A, antigen-specific Tregs, experimental autoimmune encephalomyelitis, chimeric antigen receptor, B cell antibody receptors

## Abstract

Human regulatory CD4^+^ T cells (Tregs) are potent immunosuppressive lymphocytes responsible for immune tolerance and homeostasis. Since the seminal reports identifying Tregs, vast research has been channeled into understanding their genesis, signature molecular markers, mechanisms of suppression, and role in disease. This research has opened the doors for Tregs as a potential therapeutic for diseases and disorders such as multiple sclerosis, type I diabetes, transplantation, and immune responses to protein therapeutics, like factor VIII. Seminal clinical trials have used polyclonal Tregs, but the frequency of antigen-specific Tregs among polyclonal populations is low, and polyclonal Tregs may risk non-specific immunosuppression. Antigen-specific Treg therapy, which uses genetically modified Tregs expressing receptors specific for target antigens, greatly mitigates this risk. Building on the principles of T-cell receptor cloning, chimeric antigen receptors (CARs), and a novel CAR derivative, called B-cell antibody receptors, our lab has developed different types of antigen-specific Tregs. This review discusses the current research and optimization of gene-modified antigen-specific human Tregs in our lab in several disease models. The preparations and considerations for clinical use of such Tregs also are discussed.

## Introduction

Human regulatory CD4^+^ T cells (Tregs) are a subset of adaptive lymphocytes well characterized for their immunosuppressive functions and maintenance of immunological tolerance. Tregs are broadly grouped into two categories, either natural (i.e., thymus derived) or induced (i.e., peripherally derived). Natural Tregs (nTregs) represent between 2 and 8% of CD4^+^ T cells in healthy donor peripheral blood, whereas induced Tregs can be generated by expansion of CD4^+^ T cells in the presence of TGFβ. The importance of Tregs in immune regulation and brokering tolerance has been robustly demonstrated (1–9), and expanded polyclonal Tregs are being developed for clinical applications. In this review, however, we summarize studies in our lab designed to generate antigen-specific nTregs by transduction of specific receptors.

Engineering antigen-specific T cells by gene modification has proven to be an invaluable immunological technology (10). In addition to exogenous T-cell receptors (TCRs), chimeric antigen receptors (CARs) containing single chain variable fragments (scFv) are also used to redirect polyclonal T cells to a defined specificity. We have also engineered Tregs to express antigens or antigen fragments that can be recognized by B-cell receptors, which we refer to as B-cell antibody receptors (BARs). For BARs, the scFv of the CAR is replaced with an antigen or its domain. The exogenous TCRs are generally cloned from T cells present in diseased tissue, such as tumor-infiltrating lymphocytes, pancreatic islets, or multiple sclerosis (MS) lesions and are human leukocyte antigen (HLA) (11–15). The CARs, which are synthetic molecules, are typically comprised of scFv fused to T cell co-stimulatory proteins and CD3ζ chain. The scFv portion of the CAR can be derived from phage display technology or traditional monoclonal antibody production (15–19). The antibody-derived properties of the CAR free it from HLA restriction. TCRs or CARs have traditionally been used to engineer effector T cells, predominantly CD8^+^ cytotoxic cells. The multiple design iterations, clinical successes (e.g., against melanoma and acute lymphoblastic leukemia) of TCR- and CAR gene-modified cells have been extensively reviewed by our group (20) and others (21–25). As noted above, our group and Ellebrecht et al. independently designed a novel method of engineering antigen-specific T cells with antigen domains, called BAR in our lab and called chimeric autoantibody receptor by the Payne group (26). This antigen domain targets pathogenic antibody secreting cells or their precursors with specific surface B-cell immunoglobulin (Ig) receptors (BCR). We have adapted these redirecting technologies to human Tregs with the goal of improving future Treg therapy in clinical trials.

**Figure 1:**
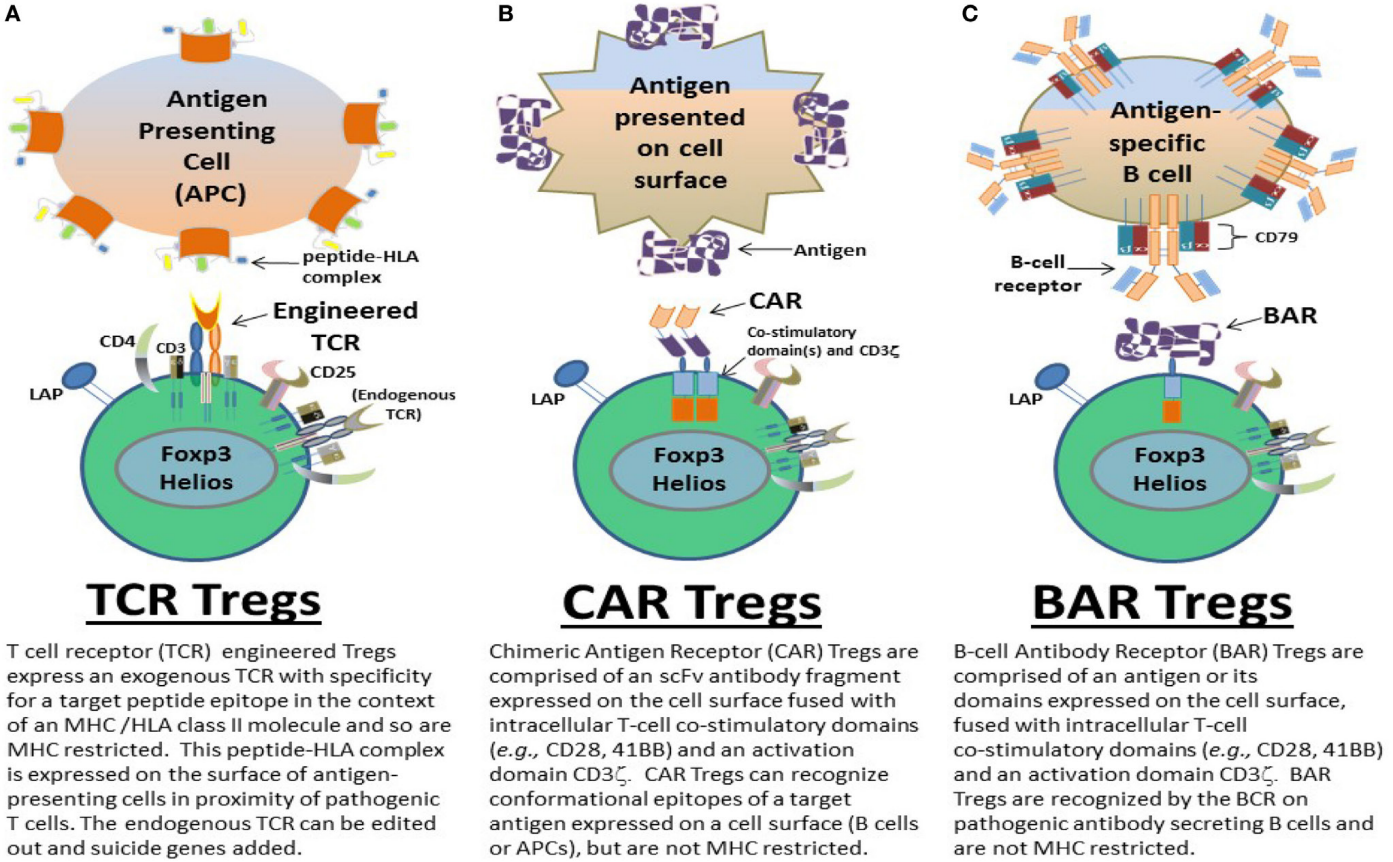
Types of gene-modified antigen-specific human regulatory CD4^+^ T cells (Tregs). Antigen-specific **(A)** T-cell receptor (TCR), **(B)** chimeric antigen receptor (CAR), and **(C)** B-cell antibody receptor (BAR) Tregs have been designed by the Scott lab as potential therapeutics to ameliorate autoimmune diseases and/or immune responses to biotherapeutics in monogenic diseases, for example. TCR, CAR, and BAR Tregs each have unique properties that can be exploited as treatments geared to the different pathophysiologies of such diseases and/or adverse immune responses. The structure and targeting moieties/cells of TCR, CAR, and BAR Tregs are briefly described and depicted.

Here, we chronologically review the development of antigen-specific human Tregs by gene modification in our lab. Specifically we describe the use of TCR (Figure 1A), CAR (Figure 1B), and BAR (Figure 1C) Treg therapy in the context of disease models for hemophilia A and MS. The important conclusions from our experiments as well as future directions and considerations for gene-modified Tregs as a therapeutic are discussed.

## nTregs Therapy: Polyclonal or Specific

Phenotypically, peripheral blood nTregs are identified by high surface expression of CD25 (IL-2 receptor α chain), low expression of CD127 (IL-7α receptor), low to negative expression of CD45RA, and expression of the transcription factors, Foxp3 and Helios. Further markers such as the Treg-specific demethylated region, glycoprotein A repetitions predominant protein (GARP), glucocorticoid-induced TNFR family-related gene (GITR), latency-associated peptide (LAP), CTLA-4, CD27, CD73, and CD39 among others also aid in nTreg identification (27–36).

FoxP3 was identified from early studies with scurfy mice, which have an idiopathic mutation in the Foxp3 gene and develop systemic multi-organ autoimmunity (37, 38). In humans, the importance of Tregs is evident in the debilitating and often fatal polyendocrinopathy, enteropathy, X-linked (IPEX) syndrome which is linked to mutations in the Foxp3 gene (39, 40). The causal link between dysfunctional Tregs and autoimmunity set the stage for using functional Tregs to treat and possibly prevent it. Indeed adoptive Treg therapy to treat animal models of autoimmunity such as experimental autoimmune encephalomyelitis (EAE), arthritis, inflammatory bowel disease, and uveitis among others has proven successful and served as proof of concept for Treg therapy translational use (41–46).

Phase 1 clinical trials using human Tregs have involved participants suffering from acute graft versus host disease (GVHD) following stem cell transplants (47) or type I diabetes (T1D) (48). For the GVHD trial, all participants were infused with umbilical cord blood-derived polyclonal Tregs. No infusion related toxicities or adverse events were reported during the trial period. However, the authors concluded that a randomized control group receiving no Tregs was necessary to properly access treatment efficacy. The T1D trial used autologous peripheral blood-derived polyclonal Tregs. This was a dose escalation phase 1 trial. Treatment efficacy was not accessed, but the escalation protocols and safety profile of this trial has led to a phase 2 trial as of 2017 (49). Further trials using polyclonal Tregs to treat lupus and GVHD from kidney transplants and liver disease have also been initiated.^1^

The majority of these clinical trials have used polyclonal Tregs. While the success of polyclonal Tregs has been promising, the amount of cells needed for infusions is large (believed to be in the 10^9^–10^10^ range) and the threat of global immune suppression is possible; indeed, one report cites viral reactivation after infusion of polyclonal Tregs (47) and tumor occurrence/recurrence is of concern given the correlation between Tregs and tumor survival (50, 51). Moreover, polyclonal human Tregs are not a homogenous population which may introduce unwanted variability and a lack of efficacy to their therapeutic potential (36, 52–54). To overcome these drawbacks, we and others believe that using antigen-specific Tregs of a defined homogenous population will require fewer cells to exert their regulatory effects and confer more localized and targeted suppression.

The occurrence of a particular antigen-specific T cell is very low, on the order of 1 in every 10^5^–10^7^ T cells (55). This greatly hinders the ability to isolate and expand such rare cells. However, in certain disease states or conditions where a target antigen or group of antigens is/are known, the clonal expansion of an antigen-specific T cell facilitates its detection and isolation by molecular methods. Such methods include tetramer-guided epitope mapping and peptide MHC microarrays (56–59). Since the TCR traditionally endows a T cell with its specificity, extracting the TCR cDNA sequence from the expanded cells and cloning it into a viral expression vector allows researchers to engineer antigen-specific T cells.

## Human Tregs Gene Modified to Express an FVIII-Specific TCR

One disease model used in our lab to study the therapeutic potential of antigen-specific Tregs is hemophilia A. Hemophilia A is an X-linked bleeding disorder caused by mutations in the factor 8 (*F8*) gene, which encodes the blood coagulation protein, FVIII. Because of its monogenic etiology, the disorder can be treated with recombinant or plasma derived FVIII replacement therapy. Unfortunately, a large subset of those receiving replacement FVIII develop an antidrug antibody response. These antibodies (referred to as “inhibitors”) can neutralize the FVIII, rendering this lifesaving treatment ineffective. Inhibitor formation requires CD4^+^ T cell help (60, 61), and is largely directed to the A2 and C2 domains of the FVIII protein.

The standard treatment for inhibitors is called immune tolerance induction (ITI). ITI consists of high dose infusions of FVIII for a period of one or more years. Although it has met with some clinical success, ITI does not work for all inhibitor cases. Thus, alternative approaches for inducing tolerance in these unsuccessful cases or preventing inhibitor responses, in the first place, are of clinical importance.

In collaboration with the lab of Dr. Kathleen Pratt, we successfully isolated, cloned and sequenced HLA-DRB1*01:01 (DR1)-restricted TCRs specific for an epitope in the C2 domain of FVIII. The TCRs were isolated from CD4^+^ T cell clones of a hemophilia A subject at different time points after clonal expansion (62).

As reported in 2015 (63), we sorted human nTregs from healthy donor peripheral blood mononuclear cells (PBMCs) and transduced them with retroviral particles encoding one of these C2 domain specific TCRs, referred to as 17195. Transduced Tregs were then sorted and expanded in the presence of antihuman CD3, autologous γ-irradiated PBMCs, and oligodeoxynucleotides (ODN). Kim et al. have shown that these ODN maintain the Treg phenotype better than inclusion of rapamycin during the critical rapid expansion period (64).

An important point with *ex vivo* expansion of human gene-modified Tregs is to determine the activation status of the Tregs during and/or at the end of the expansion. Initial *in vitro* activation of sorted Tregs for 3–5 days is necessary for retro- or lentiviral gene transfer, followed by large-scale expansion for 10–12 days with IL-2, but without TCR or anti-CD3 stimulation. This expansion step generally can be repeated for up to two more cycles. In most cases, successfully expanded gene-modified Tregs do not retain their activation status due to the long-term expansion conditions without cognate/specific antigen (e.g., TCR) or anti-CD3 stimulation. Nonetheless, *in vitro* confirmation of gene-modified Treg activation with specific antigen is mandatory before testing these Tregs *in vivo*. Such confirmation provides a functional estimation of the Treg responsiveness. For this, surface expression of GARP, LAP, and CD25 as well as the induction of Foxp3 and Helios are analyzed by flow cytometry at 24–48 h post *in vitro* activation with cognate antigen and PBMCs (63, 65).

Tregs expressing the 17195 TCR proliferated in an antigen-specific manner and, importantly, maintained their Treg phenotype. Moreover, as mentioned above, these cells upregulated the Tregs markers Foxp3, Helios, GARP, LAP, and CD25 when stimulated with specific peptide. This phenotypic response was mirrored by the fact that they were able to prevent FVIII-specific effector cells from proliferating, as demonstrated in an *in vitro* suppression assay. Of clinical note, these Tregs also robustly diminished FVIII antibody production in splenocytes of FVIII-immunized HLA DR1 transgenic hemophilic mice *in vitro* and could prevent anti-FVIII formation *in vivo* in a xenogeneic transfer system.

## Human Tregs Gene Modified to Express an FVIII-Specific CAR

Following the promising results and lessons gleaned from the FVIII-specific TCR gene-modified Tregs, we sought to design a FVIII-specific CAR Treg. CAR Tregs would allow us to test, without HLA restriction, the inhibition of both FVIII-specific antibody production and effector T cell proliferation. In collaboration with the lab of Drs. Anja Schmidt and Christoph Königs, Yoon et al. published results of human FVIII-specific CAR Tregs, referred to as ANS8 CAR Tregs (65). The human scFv region of the CAR was isolated by phage display and confirmed specific for the A2 domain of FVIII by competitive ELISA using known monoclonals against this domain (66). ANS8 CAR Tregs proliferated in response to FVIII and also concomitantly upregulated Foxp3 expression. These CAR Tregs suppressed the proliferation of FVIII-specific effector T cells. Moreover, these CAR Tregs also exhibited bystander suppression as they were able to prevent the proliferation of HLA DR2-restricted T effector cells specific for a myelin basic protein (MBP) peptide in the presence of appropriate antigen-presenting cells. Strikingly, when tested *in vivo*, ANS8 CAR Tregs were able to prevent FVIII antibody titers *prophylactically*, similar to TCR-transduced (17195) Tregs. The prevention of the anti-FVIII response was sustained up to 8 weeks despite the rejections of the transferred human Tregs in immunocompetent mice. This emphasized the potency of the ANS8 CAR and TCR-transduced Tregs and has prompted us to design *in vivo* therapeutic protocols for FVIII antibody prevention.

## Human Tregs Gene Modified to Express a Bar Specific for FVIII Inhibitors

To test whether engineered Tregs could directly suppress B cells, we designed a third engineered T cell model that would express antigen and would directly interact with specific B cells *via* their BCR. Thus, our latest gene-modified human Tregs are engineered to express either the immunodominant A2 or C2 domains of FVIII, fused to T cell co-stimulatory and signaling domains, so called “BAR” for B-cell antibody receptor. It has been shown in animal models of autoimmunity and suggested in IPEX patients that Tregs may be able to directly suppress pathogenic B cells (67–70). In light of these studies, we hypothesized that BAR engineered Tregs directly suppress FVIII-specific B cells *via* interaction with their BCR and may possibly suppress other FVIII-specific effector T cells co-localized in the local milieu.

Zhang et al. (71) in our lab showed that A2 and C2 BAR Tregs maintained Treg-specific markers, including Foxp3 and Helios, after long-term expansion *in vitro*. Importantly, we showed that these BAR Tregs also potently suppressed FVIII antibody formation *in vitro* and *in vivo* from FVIII-immunized hemophilic mice, thus providing a third model of specific Tregs. The mechanism of this suppression is discussed below.

## Human Tregs Gene Modified to Express an MBP-Specific TCR

Another important disease studied in our lab is MS. We employ an EAE mouse model for MS. MS is a debilitating autoimmune disorder where effector T cells mediate the attack and destroy the myelin sheath of the central nervous system (CNS). This destruction results in relapsing/remitting symptoms or progressive paralysis, which could result in death in its most severe cases. The etiology of MS is unknown, but certain genetic and environmental factors may play a role (72–75). Current treatment options include immunosuppressive drugs, β-interferon, or Copaxone, a random amino acid copolymer (76–78). Recently, treatment with B-cell depleting antibodies such as ocrelizumab and rituximab (79–83), has been used to relieve symptoms, but their side effects can be severe and also can lead to global immunosuppression (84, 85). Better treatment options thus are clearly warranted. We believe that antigen-specific Tregs targeting CNS antigens implicated in MS can be such an option.

We engineered a construct to express a TCR sequence provided by Dr. Kai Wucherpfennig, who isolated the TCR from an autoreactive CD4^+^ T cell clone of an MS patient. This TCR, referred to as Ob2F3 (86–88), was specific for MBP epitope 85-99 and was HLA DR15 (“DR2”) restricted. PBMC obtained from normal healthy donors were FACS-purified for nTregs, as we had done in the FVIII project, and transduced with the Ob2F3 TCR. These expanded, now MBP-specific, Tregs not only suppressed MBP-specific T-cell proliferation and cytokine production but also they could suppress FVIII-specific responses *in vitro* when both MBP and FVIII peptides were present. Remarkably, Ob2F3 TCR Tregs were also able to reduce myelin oligodendrocyte glycoprotein (MOG 35-55)-induced EAE symptoms in HLA DR2-transgenic mice. This was important because it confirmed that Tregs of one specificity (MBP) could exert bystander suppression of T effectors of another specificity (MOG), presumably in the local milieu. We found that these Ob2F3 TCR Tregs migrated in greater numbers to the CNS than non-specific Tregs and reduced the perivascular infiltrates in the spinal cord. This xenogeneic suppression validates the potency of antigen-specific engineered Tregs.

## Mechanisms of Suppression

Understanding the suppression mechanism behind our gene-modified human Tregs is also actively being pursued. Although it has been shown that Tregs have a diverse repertoire of suppression strategies both contact independent (contactless) and contact dependent (89–95), how these specifically modified Tregs suppress target cells is currently unresolved. While we know that bystander suppression could occur *in vitro* and *in vivo*, it was not clear whether cell-to-cell contact was needed. To investigate contactless and contact-driven mechanisms, our lab used a modified transwell developed by Dr. Kim that consisted of “heat-drilling” holes between microtiter wells so that liquid (and presumably effector suppressive molecules) could mix in the interwell space, dubbed the de-cellularized zone. We found that suppression of effector T-cell proliferation only occurred when specific Tregs *and* specific effector T cells were present together in the adjacent well (96).

We know that both effector and regulatory T cells need IL-2 to grow (97, 98). When we examined Stat5 phosphorylation kinetically, we found that antigen-stimulated effector CD4^+^ T cells produced and responded to IL-2 with Stat5 phosphorylation starting at 8 h, but that Tregs alone showed minimal Stat5 phosphorylation even at 72 h. However, when cocultured together, Treg Stat5 phosphorylation started as early as 8 h, at which time the CD4^+^ T cell effector response to IL-2 decreased dramatically. These results suggest that Tregs “co-opt” IL-2 from effector T cells and that a contact-dependent process was initiated with the production of more (long-acting) suppressive moieties.

To understand potential BAR Treg suppression mechanisms in our hemophilia A model, we designed a series of B and T cell coculture assays. Briefly, splenic B and T cells were isolated from A2 and C2 BAR Treg treated or non-specific control BAR Treg-treated FVIII-immunized hemophilic mice. T cells, isolated from A2 and C2 BAR Treg treated mice, were able to cooperate and stimulate antibody formation with B cells from control mice. However, B cells isolated from A2- and C2-tolerized mice failed to be stimulated for anti-FVIII antibody production by control T cells. These observations strongly suggest that A2 and C2 BAR Tregs tolerized the B cell compartment while sparing that of T cells. Further experiments assessing whether A2 and C2 BAR domains are taken up by specific B cells (as exosomes or by trogocytosis?) or whether this tolerization of different compartment has a kinetic component (i.e., T cells become tolerized at a later time point) are underway.

To facilitate further mechanistic studies, we are reversing our trajectory back into murine systems. Our human Tregs are eventually rejected by the mouse immune system so trafficking studies, adoptive transfers and re-challenge experiments are not feasible. In addition, the use of knockout murine cells will aid in completing the mechanistic picture of gene-modified Tregs. These studies are in progress.

Please see Table 1 for summary of results.

**Table 1:** Types of antigen-specific human Tregs used in the Scott lab.

Gene-modified hTreg	Specificity/target antigen	Disease model	Results
17195 T-cell receptor (TCR) Tregs	Human leukocyte antigen (HLA) DR1-restricted FVIII epitope (C2191–2210)	Hemophilia A	Expanded in an antigen-specific manner and maintained Treg phenotype following long-term *in vitro* expansion Suppression of specific T effectors *in vitro* Suppressed FVIII-specific antibody production *in vitro* and *in vivo* across a xenogeneic barrier Bystander suppression in the local milieu

ANS8 chimeric antigen receptor (CAR) Tregs	A2 domain of FVIII	Hemophilia A	Expanded in an antigen-specific manner and maintained Treg phenotype following long-term *in vitro* expansion Suppression of specific T effectors *in vitro* Suppressed FVIII-specific antibody production *in vitro* and *in vivo* across a xenogeneic barrier Bystander suppression in the local milieu

A2 and C2 B-cell antibody receptor (BAR) Tregs	B-cell receptors specific for A2 or C2 domains of FVIII	Hemophilia A	Expanded in an antigen-specific manner and maintained Treg phenotype following long-term *in vitro* expansion Suppressed FVIII-specific antibody production *in vitro* and *in vivo* across a xenogeneic barrier Bystander suppression in the local milieu Direct suppression of FVIII-specific B cells

OB2F3 TCR Tregs	HLA DR15-restricted myelin basic protein epitope (MBP 85-99)	Multiple sclerosis [experimental autoimmune encephalomyelitis (EAE)]	Suppressed MOG specific T cells *in vitro* Suppressed MOG peptide induced EAE across a xenogeneic barrier Trafficked to brain and spinal cord

*The disease models in which they are tested and related results are listed and summarized, respectively*.

## Future Directions and Considerations for Gene-Modified Human Tregs

T-cell receptor, CAR, and BAR Treg therapy all provide distinct advantages and (minor) disadvantages as therapeutics. All of these Tregs, while highly specific, can exhibit bystander suppression in the local milieu as demonstrated by their ability to suppress inhibitor formation to the entire FVIII protein *in vitro* and *in vivo*, despite being specific for a single domain or peptide epitopes. TCR gene-modified Tregs allow for targeting specific peptides presented by APC to pathogenic effector cells. The TCR also allows for the physiological activation and regulation of the Tregs. However, the HLA restriction of TCR limits its utility to recipients sharing those HLA class II antigens. This is not as serious with MS, for example, as there is linkage to HLA DR2 (99). However, strong linkage to HLA has not been observed in hemophilia A (100). Nonetheless, there are five to seven most common DR phenotypes in North American Caucasians; conceivably, one could clone the V genes from the T cells of patients with these HLA DRs to create a repertoire of TCRs. Thus, screening for HLA or engineering TCRs *de novo* for each recipient is feasible today.

Chimeric antigen receptor gene-modified Tregs have the advantage of being HLA unrestricted. This greatly increases the universality of their usage as a therapeutic in all patients. These Tregs, like the TCR-transduced Tregs, can exhibit bystander suppression but need to recognize conformational domains in the target antigen. This is likely to occur in the context of cell surfaces, either dendritic or endothelial cells or specific B cells, before uptake. The scFv we have used was obtained by phage display (65). Thus, further scFvs against other domains of FVIII can readily be produced.

B-cell antibody receptor Tregs represent a novel approach for engineering gene-modified antigen-specific cells; these too are not HLA-restricted and only require that specific B cells can bind *via* their surface Ig receptors to the domains expressed on the Tregs. Originally, our lab envisioned this approach for targeting inhibitors in hemophilia A or responses to biotherapeutics in monogenic diseases, but they also can be designed to target pathogenic antibodies in autoimmunity or antidrug antibodies (101, 102). An issue with BARs Tregs (or BAR CD8 killer T cells) is that circulating antibodies may bind to the BAR Treg epitope domains and either neutralize their activity or cause tonic signaling to drive an exhausted phenotype. While a concern, we think this is unlikely since we have found that antibody crosslinking of the BAR can, in some instances, trigger Treg proliferation. In addition, plasmapheresis could be used to remove the circulating antibodies if needed, but these may not possess as high an affinity for the BAR as the isotype-switched memory B cells.

Much remains to be discovered regarding specific Treg suppression mechanisms. We already know that, aside from bystander suppression which occurs locally, the contiguous presence of effector T cells and Tregs can lead to enhanced suppressive activity and contactless suppression of other T cells. This is in part due to the fact that effector cells require much higher amounts of IL-2 to maintain proliferation compared to Tregs, which acquire IL-2 locally and rapidly phosphorylate Stat5 downstream of CD25. How this process activate the Tregs to produce suppressive moieties is unknown but under investigation.

How Tregs modulate antibody formation is not clear. Obviously, suppression of effector (helper) T cell activation is involved. In a preliminary experiment, culture of T and B cells from BAR Treg-tolerized hosts suggests that B cells may be directly targeted (at least by BAR Tregs). We have no evidence at present for direct toxicity of BAR Tregs on B cells, but this remains an open question since human CD4^+^ T cells can be cytotoxic (103, 104).

A major concern of any gene-modified cellular therapy is safety. Fortunately, technologies such as inducible suicide genes can be applied to gene-modified Tregs (105). For example, this technology would be a protection in the unlikely event in which bystander suppression led to any unintended sequelae. In addition, we would like to create specific Tregs from generic, “off-the-shelf” unrelated donors. While such cells would possess endogenous TCRs and foreign HLA, we plan to apply techniques, such as CRISPR/Cas9 or TALENs (106, 107), to engineer out the endogenous TCRs so that these Tregs would only possess the transduced TCR, CAR, or BAR and would not cause GVHD disease. In addition, deleting HLA class II would also prevent their rejection by an immunocompetent host. Expansion of Tregs and their stable phenotype are also important concerns in advancing gene-modified Treg therapy. It is estimated that a minimal dosage of 1–3 × 10^6^/kg of Treg may be needed for therapy efficacy (108). As mentioned, the ODN protocol has proven effective in maintaining the human Treg phenotype and function throughout the necessary expansion phase.

Designing treatments for an ongoing and established autoimmune diseases can pose a significant hurdle. Typically, autoimmune patients first visit their physicians after disease symptoms have appeared. The dysregulation and disruption of self-tolerance leading to diabetes, lupus, or MS, for example, typically can occur over an extended asymptomatic or subclinical time period. However, predictive measures in addition to family history are being developed. Therefore, antigen-specific Treg therapy could be administered to these patients to prevent pathological damage. Our data suggest we may even be able to attenuate symptoms once they arise. While there can be multiple (and unknown) target antigens in autoimmune diseases, we have shown in our EAE/MS model that gene-modified antigen-specific Treg therapy can exhibit bystander tolerance for other antigens in the local milieu.

**Figure 2:**
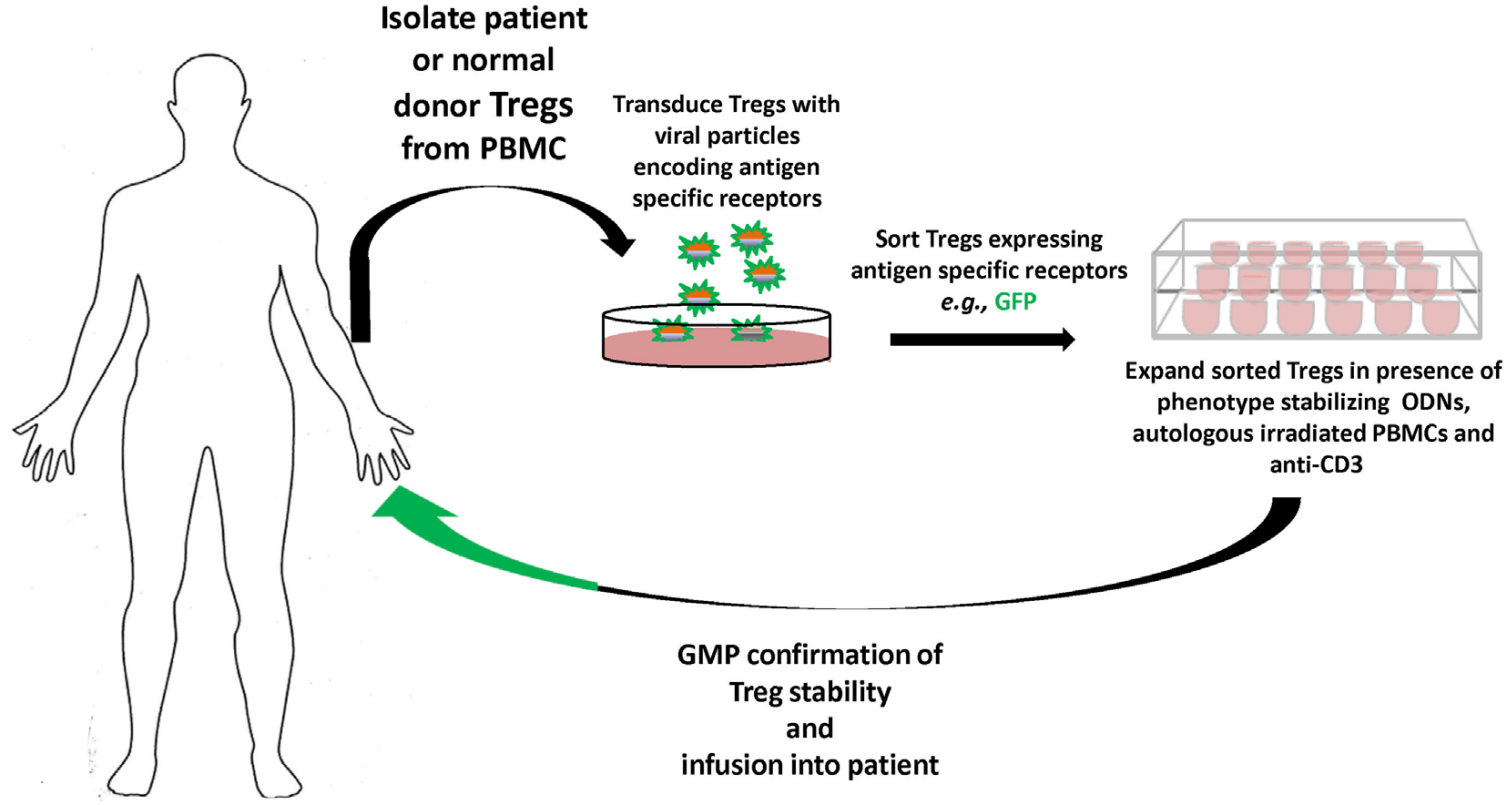
Overview of gene-modified antigen-specific human Treg therapy. Patient (or normal donor) blood is collected, and Tregs are sorted from buffy coat, and virally transduced to express specific receptors [T-cell receptor (TCR), chimeric antigen receptor, or B-cell antibody receptor]. The antigen-specific Tregs are then sorted and expanded in the presence of autologous peripheral blood mononuclear cells (PBMCs), anti-CD3, and oligodeoxynucleotides (ODN), which stabilize Treg functional characteristics during expansion. The antigen-specific Tregs that meet robust GMP standards and Treg phenotype are then infused back into the patient tracking of the Tregs *in vivo* can be performed by deuterium labeling or GFP expression. Safety constructs that trigger the ablation or death of the infused antigen-specific Tregs can also be integrated, and gene editing by CRISPR/Cas9, e.g., used to remove endogenous TCRs or MHC to avoid graft versus host disease or rejection, respectively, of generic donor T cells.

In terms of responses to biotherapeutics, as in hemophilia A or monogenic diseases like Pompe’s, the specific antigens are known, as is family history; hence, gene-modified antigen-specific human Tregs can become viable therapies prophylactically, as well as therapeutically. Our results suggest that antigen-specific TCR, CAR, and BAR Tregs each have distinct advantages as therapeutics and, thus, cultivation of each is necessary. Current progress toward good manufacturing practice and economy of scales are now being optimized. The future promise of gene-modified specific human Tregs therapeutics is quickly becoming today’s reality (Figure 2).

## Author Contributions

PA and DS: writing organization of manuscript citations and creation of figures. YK, A-HZ, and JY: proofreading and discussion.

## Conflict of Interest Statement

The authors declare that the research was conducted in the absence of any commercial or financial relationships that could be construed as a potential conflict of interest.

## Footnotes

^1^Available from: https://clinicaltrials.gov/ct2/results?term=adoptive+treg+therapy&Search=Search
